# Identification of a series of hair-cell MET channel blockers that protect against aminoglycoside-induced ototoxicity

**DOI:** 10.1172/jci.insight.145704

**Published:** 2021-04-08

**Authors:** Emma J. Kenyon, Nerissa K. Kirkwood, Siân R. Kitcher, Richard J. Goodyear, Marco Derudas, Daire M. Cantillon, Sarah Baxendale, Antonio de la Vega de León, Virginia N. Mahieu, Richard T. Osgood, Charlotte Donald Wilson, James C. Bull, Simon J. Waddell, Tanya T. Whitfield, Simon E. Ward, Corné J. Kros, Guy P. Richardson

**Affiliations:** 1 Sussex Neuroscience and; 2 Sussex Drug Discovery Centre, School of Life Sciences, and; 3 Global Health and Infection, Brighton and Sussex Medical School, University of Sussex, Brighton, United Kingdom.; 4 Bateson Centre and Department of Biomedical Science, and; 5 Information School, University of Sheffield, Sheffield, United Kingdom.; 6 Department of Biosciences, College of Science, Swansea University, Swansea, United Kingdom.; 7 Medicines Discovery Institute, Cardiff University, Cardiff, United Kingdom.

**Keywords:** Neuroscience, Therapeutics, Drug screens, Mouse models

## Abstract

To identify small molecules that shield mammalian sensory hair cells from the ototoxic side effects of aminoglycoside antibiotics, 10,240 compounds were initially screened in zebrafish larvae, selecting for those that protected lateral-line hair cells against neomycin and gentamicin. When the 64 hits from this screen were retested in mouse cochlear cultures, 8 protected outer hair cells (OHCs) from gentamicin in vitro without causing hair-bundle damage. These 8 hits shared structural features and blocked, to varying degrees, the OHC’s mechano-electrical transducer (MET) channel, a route of aminoglycoside entry into hair cells. Further characterization of one of the strongest MET channel blockers, UoS-7692, revealed it additionally protected against kanamycin and tobramycin and did not abrogate the bactericidal activity of gentamicin. UoS-7692 behaved, like the aminoglycosides, as a permeant blocker of the MET channel; significantly reduced gentamicin–Texas red loading into OHCs; and preserved lateral-line function in neomycin-treated zebrafish. Transtympanic injection of UoS-7692 protected mouse OHCs from furosemide/kanamycin exposure in vivo and partially preserved hearing. The results confirmed the hair-cell MET channel as a viable target for the identification of compounds that protect the cochlea from aminoglycosides and provide a series of hit compounds that will inform the design of future otoprotectants.

## Graphical abstract

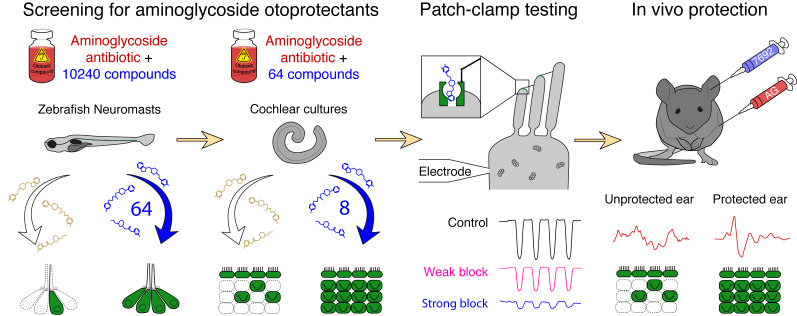

## Introduction

Aminoglycoside antibiotics offer an effective treatment against life-threatening infections, including sepsis and tuberculosis (1, 2). They do, however, cause dose-related nephro- and ototoxicity (3, 4). Whereas damage to the kidneys is reversible (5), some degree of permanent hearing loss has been reported in approximately 20% of patients treated with aminoglycosides (6, 7). Vestibular dysfunction is also not uncommon and may even be more frequent (8, 9). The clinical use of these antibiotics will continue until further drugs are developed that are affordably priced, available worldwide, and equally effective against infections currently treated with aminoglycosides. Finding methods to prevent the associated hearing loss is therefore critical.

Most attempts to identify compounds that could be coadministered with aminoglycosides to provide otoprotection without diminishing bactericidal activity have focused on testing antioxidants and antiapoptotic agents (10–19). Other attempts have screened libraries of FDA-approved drugs (20) or libraries of molecules that interact with ion channels (21). Thus far, there has been only 1 unbiased screen of a moderately large and diverse chemical library (22), a study in which 10,960 compounds in the Chembridge Diverse E set were screened at 10 μM for ability to protect zebrafish lateral-line hair cells against short-term exposure to a high concentration of neomycin (200 μM). This screen identified 2 compounds, PROTO-1 and PROTO-2, both of which protected extrastriolar hair cells in mature mouse utricles from neomycin in vitro (22). Subsequent phenotypic optimization of PROTO-1 led to the development of ORC-13661, an orally available derivative that preserves hearing in rats treated with the aminoglycoside amikacin (23) and prevents hair-cell death in mouse cochlear cultures exposed to either the ototoxic anticancer reagent cisplatin or the aminoglycoside gentamicin (24). Furthermore, ORC-13661 is a high-affinity permeant blocker of the mechano-electrical transducer (MET) channel, an established route of aminoglycoside entry into hair cells (25–28).

The clinical effectiveness of ORC-13661 remains to be determined, and it would be advantageous if a broader repertoire of potential protectants was available. Neomycin, used in the zebrafish Diverse E library screen (22), and gentamicin, widely used in neonatal intensive care units (29), differ in the way in which they kill hair cells (30). Furthermore, the concentration of neomycin (200 μM) used in the zebrafish Diverse E screen (22) is in considerable excess of the endolymphatic gentamicin concentration (~2 μM) measured in rats with plasma levels comparable to those of patients treated with this drug (31). Although high aminoglycoside concentrations provide a time-saving advantage in moderate-to-high throughput screens, one may miss compounds effective against lower, but nonetheless clinically relevant, concentrations of aminoglycosides.

In light of these concerns, a Life Chemicals Diversity Set of compounds covering a broad range of chemical space was screened using zebrafish larvae in a pipeline that used 2 aminoglycosides at lower concentrations and involved 3 sequential selections: the first with zebrafish and neomycin, the second with zebrafish and gentamicin, and the third with mouse cochlear cultures and gentamicin. The zebrafish screens provided a high yield of compounds (64 in total) that protected against both neomycin and gentamicin, 8 of which provided protection against gentamicin in mouse cochlear cultures. These hits all interact with the MET channel to varying degrees, and suggest this hair-cell entry route for aminoglycosides is a viable target for the future development of otoprotectants.

## Results

### Screening for protectants with zebrafish larvae and mouse cochlear cultures.

**Figure 1:**
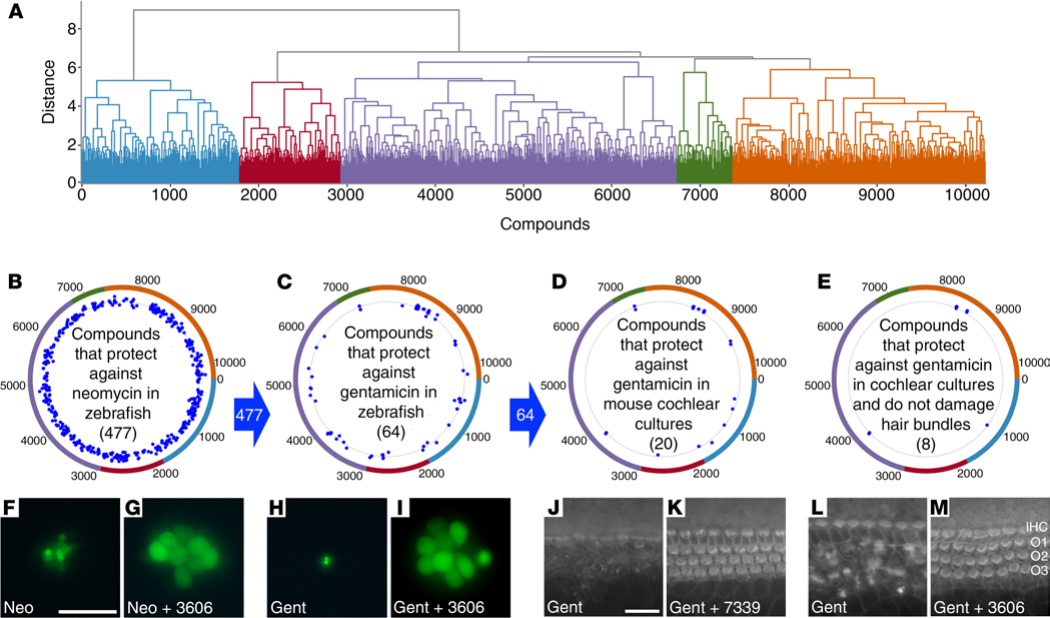
Screen of 10,240 Life Chemicals Diversity compounds in zebrafish larvae and mouse cochlear cultures. (**A**) Dendrogram of compounds grouped by broadly related structure into 5 clusters identified by color. (**B**–**E**) Polar scatterplots annotated with dendrogram clusters. Blue dots show “hit” compounds in each cluster obtained in each screen (see Supplemental Figure 1). Blue arrows between the plots indicate compounds passing the previous screen were used in the next. (**F**–**M**) Representative examples of neuromasts (**F**–**I**) and cochlear cultures (**J**–**M**) treated with antibiotic alone (**F**, **H**, **J**, and **L**) or antibiotic in the presence of one of the hit compounds (**G**, **I**, **K**, and **M**). Neuromasts (**F**–**I**) were labeled with YO-PRO-1 prior to incubation with neomycin alone (**F**), neomycin and UoS-3606 (**G**), gentamicin alone (**H**), or gentamicin and UoS-3606 (**I**). Cochlear cultures (**J**–**M**) were labeled with Texas red phalloidin after treatment with gentamicin alone (**J** and **L**) and gentamicin with UoS-7339 (**K**) or UoS-3606 (**M**). Hair bundles in **K** show damage; those in **M** are normal. Scale bars: 20 μm (**J**–**M**).

Initially, 10,240 compounds from the Life Chemicals Diversity Set (Figure 1A) were screened at a concentration of 25 μM for ability to protect hair cells in the lateral-line organs of zebrafish larvae from exposure to 6.25 μM neomycin. This approach (see Supplemental Methods; supplemental material available online with this article; https://doi.org/10.1172/jci.insight.145704DS1) yielded 477 compounds (Figure 1B), which were retested at 50 μM for their ability to protect the zebrafish hair cells from death caused by exposure to 10 μM gentamicin (Figure 1C). From these 2 screens, 64 compounds were identified (Figure 1C) that protected zebrafish lateral-line hair cells against both neomycin and gentamicin. These 64 compounds were then tested at 50 μM for their ability to protect outer hair cells (OHCs) in mouse cochlear cultures from exposure to 5 μM gentamicin. Based on qualitative assessments, 20 compounds were identified that protected mouse cochlear OHCs against gentamicin-induced cell death (Figure 1D), 8 of which protected hair cells without causing overt signs of hair-bundle damage (Figure 1E). Examples of neuromasts and cochlear cultures treated with aminoglycoside alone or aminoglycoside in the presence of a typical hit compound at each stage of this screen are shown in Figure 1, F–M. The chemical identifiers of all compounds tested and those selected at each step in the screen can be viewed in the interactive polar scatterplots (see https://sussex-neuroscience.github.io/Identification-of-a-series-of-hair-cell-MET-channel-blockers/Supplemental%20Data%20Polar%20Scatterplot.html and instructions in Supplemental Figure 1). Given that changes in hair-bundle structure are associated with resistance to aminoglycoside toxicity (32), the 8 compounds (Figure 1E) that protected OHCs without causing overt signs of hair-bundle damage (Figure 1, L and M) were selected and characterized further.

### Characterization of identified otoprotectants.

Further characterization of the protectants identified above included (a) quantitating OHC numbers in cochlear cultures treated with gentamicin together with the compound, (b) ascertaining whether the compound is cytotoxic in vitro at higher concentrations, and (c) determining whether the compound blocks either the basolateral potassium current (*I*_K,neo_) or the MET channel of early postnatal OHCs.

**Figure 2:**
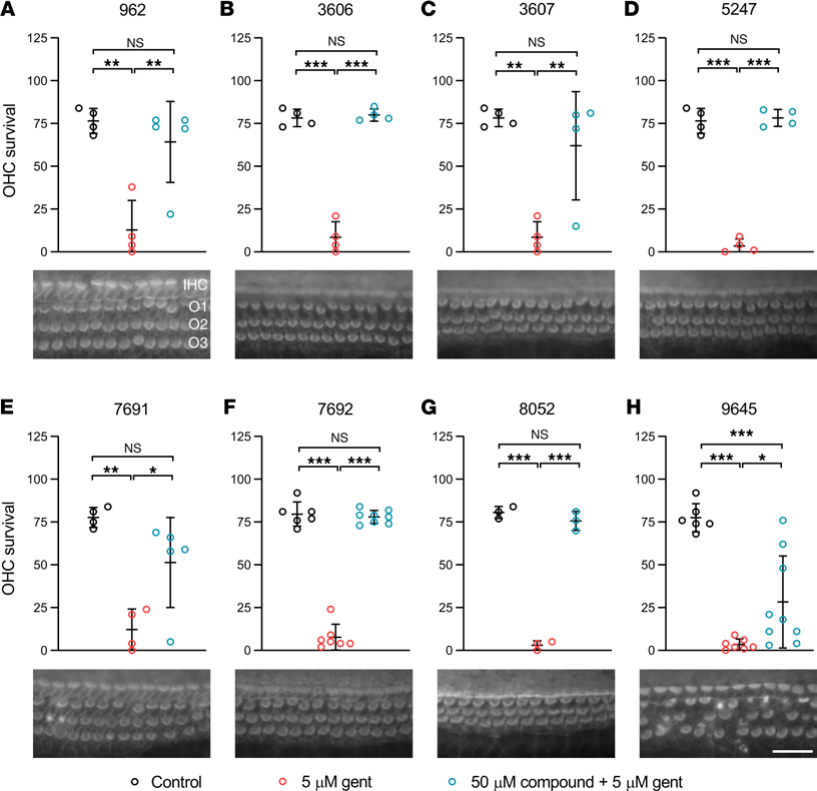
Compounds protecting mammalian cochlear hair cells from gentamicin without visible damage to the hair bundles. (**A**–**H**) Graphs showing the numbers of surviving OHCs in a region of interest (ROIs) 20% from the basal end of the cochlea in cultures exposed to low-serum medium (LSM), LSM with 5 μM gentamicin, or LSM with 5 μM gentamicin and 50 μM of (**A**) UoS-962, (**B**) UoS-3606, (**C**) UoS-3607, (**D**) UoS-5247, (**E**) UoS-7691, (**F**) UoS-7692, (**G**) UoS-8052, and (**H**) UoS-9645. DMSO at 0.5% in all conditions. Each compound was tested on the following number of independent occasions alongside a control and 5 μM gentamicin condition: UoS-962 (*n* = 4), UoS-3606 (*n* = 4), UoS-3607 (*n* = 4), UoS-5247 (*n* = 4), UoS-7691 (*n* = 4), UoS-7692 (*n* = 6), UoS-8052 (*n* = 3), and UoS-9645 (*n* = 6); additionally, in some instances compounds were tested twice within 1 experiment. One-way ANOVA with Tukey’s multiple-comparison test revealed all compounds were protective, with numbers of OHCs in the presence of test compounds and gentamicin significantly different from cultures exposed to gentamicin alone. Numbers of OHCs in cultures incubated with gentamicin and UoS-9645 were significantly lower than those in cultures exposed to LSM alone. Symbols represent individual replicates. Each symbol represents numbers of OHCs in a mid-basal ROI from 1 culture. Error bars show standard deviation of the mean (SDM). *P < 0.05; **P < 0.01; ***P < 0.001; NS, no significant difference. Representative images of the ROI from cultures exposed to LSM with 5 μM gentamicin and 50 μM of each compound show the protection offered, with phalloidin-stained hair bundles clearly visible. Scale bar: 25 μm.

**Figure 3:**
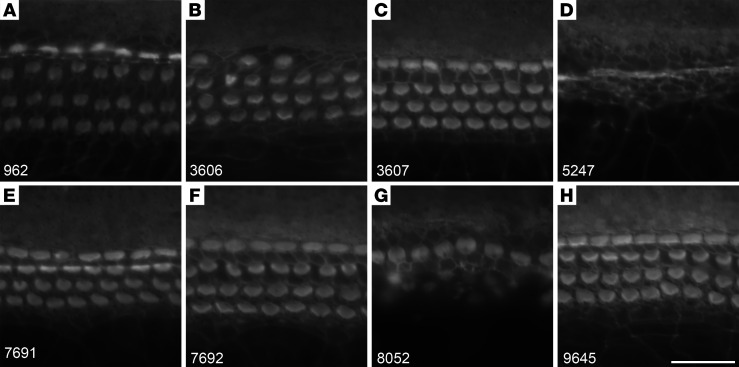
UoS-5247 and UoS-8052 are cytotoxic at a concentration of 100 μM. (**A**–**H**) Representative images from the mid-basal ROI of Texas red phalloidin-stained cultures that were exposed to 100 μM of UoS-962 (**A**), UoS-3606 (**B**), UoS-3607 (**C**), UoS-5247 (**D**), UoS-7691 (**E**), UoS-7692 (**F**), UoS-8052 (**G**), and UoS-9645 (**H**) alone for 48 hours. UoS-5247 (**D**) and UoS-8052 (**G**) show widespread cell death. Images are representative of *n* = 2 experiments. UoS-8052 was selectively toxic to OHCs, whereas UoS-5247 killed both inner and outer hair cells. Scale bar: 25 μm.

Analysis of the numbers of OHCs surviving 48 hours of exposure to 5 μM gentamicin in the presence of 50 μM of each of the 8 hit compounds (Figure 2) revealed that all were protective, with the numbers of OHCs being significantly higher than those in cultures exposed to 5 μM gentamicin alone. The protection provided by UoS-9645 was, however, inconsistent (Figure 2H), and OHC numbers were significantly lower (*P* < 0.001) than those in control cultures not exposed to gentamicin. When these 8 compounds were tested at a concentration of 100 μM in the absence of gentamicin for 48 hours (Figure 3), UoS-5247 (Figure 3D) caused a complete loss of basal-coil hair cells, and UoS-8052 (Figure 3G) caused a substantial loss of OHCs.

**Figure 4:**
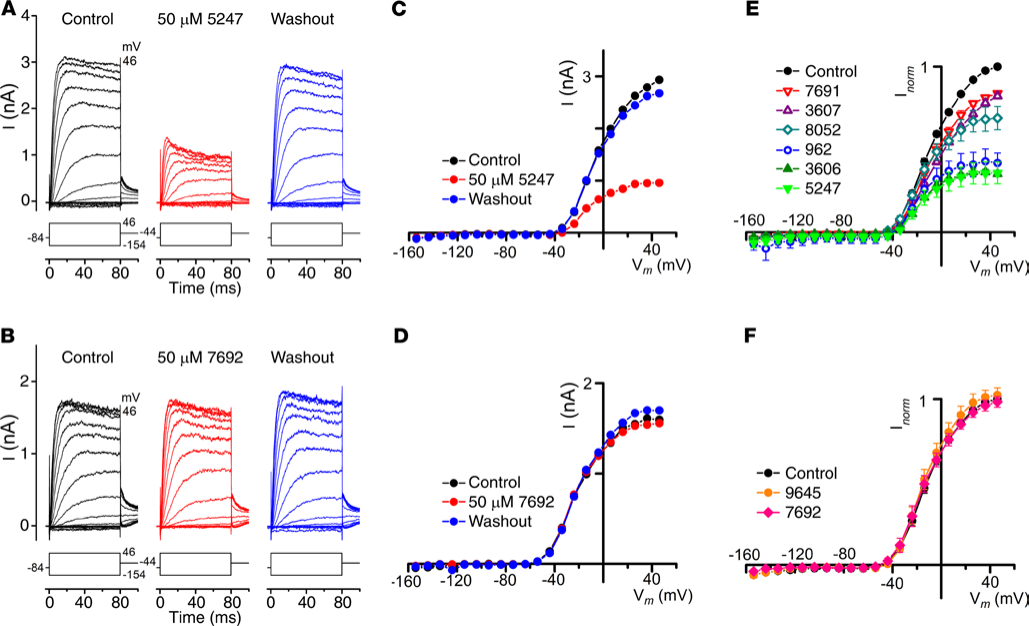
Compounds UoS-7692 and UoS-9645 do not block *I*_K,neo_. (**A** and **B**) Basolateral currents measured from mid-basal coil OHCs in response to a series of voltage steps from a holding potential of –84 mV before, during, and after extracellular exposure to 50 μM UoS-5247 (**A**) and 50 μM UoS-7692 (**B**). Schematic representation of the voltage-step protocol is shown below each of the current traces. Cell capacitances were 6.8 pF (**A**) and 7.0 pF (**B**). (**C** and **D**) Current-voltage curves measured from cells exposed to 50 μM UoS-5247 (**C**) and UoS-7692 (**D**) before, during, and after compound superfusion. (**E** and **F**) Average steady-state current-voltage curves of the 8 protective compounds at 50 μM. Currents normalized to steady-state control current at +46 mV for each cell. Cell numbers: UoS-962 (*n* = 2); UoS-3606 (*n* = 2); UoS-3607 (*n* = 2); UoS-5247 (*n* = 5); UoS-7691 (*n* = 4); UoS-7692 (*n* = 4); UoS-8052 (*n* = 5); UoS-9645 (*n* = 5).

OHCs in early postnatal mice express a voltage-dependent basolateral potassium current, *I*_K,neo_ (33), and depolarization with high extracellular K^+^ protects OHCs in cochlear cultures from aminoglycosides (21). A block of *I*_K,neo_ over the duration of the protection assay (48 hours) could cause a degree of depolarization, reducing the driving force for aminoglycoside entry into hair cells via the MET channel and thereby causing protection. To determine whether any of the 8 compounds interact with *I*_K,neo_, basolateral potassium currents were recorded in response to voltage-steps from –154 to +46 mV before, during, and after superfusion with 50 μM of each compound (Figure 4). Examples of recordings obtained with 2 of the compounds are shown in Figure 4, A and B. UoS-5247 reversibly reduced the size of *I*_K,neo_ during superfusion, with the currents recovering after washout with control solution (Figure 4, A and C), whereas UoS-7692 was without effect (Figure 4, B and D). Average current-voltage curves, normalized to the control current at +46 mV for each cell, showed that 6 of the compounds blocked *I*_K,neo_ to a variable extent (Figure 4E) and that 2 did not (Figure 4F).

**Figure 5:**
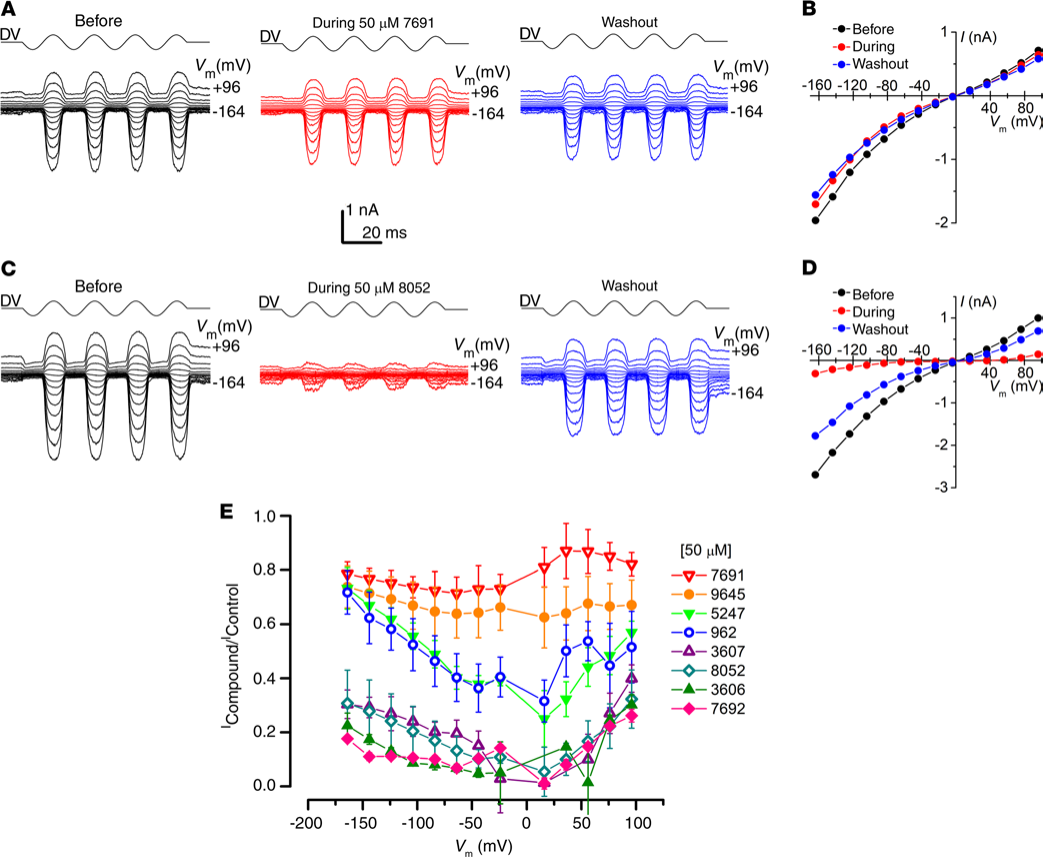
All 8 hit compounds block the MET channel to varying degrees. (**A**) MET currents recorded from an OHC before, during, and after extracellular exposure to 50 μM UoS-7691. Currents were recorded at membrane potentials ranging from –164 to +96 mV with channel opening and closing achieved by a sinusoidal stimulus delivered to the fluid jet (driver voltage, DV). (**B**) Current-voltage curves before, during, and after extracellular exposure to 50 μM UoS-7692 derived from currents shown in **A**. Cell capacitance was 8.2 pF. (**C**) MET currents from another OHC before, during, and after exposure to 50 μM UoS-8052. (**D**) Current-voltage curves derived from the currents shown in **C**. Cell capacitance was 7.0 pF. (**E**) Average fractional block curves (mean ± SEM) for all 8 compounds at 50 μM. Cell numbers: UoS-962 (*n* = 7); UoS-3606 (*n* = 4); UoS-3607 (*n* = 4); UoS-5247 (*n* = 4); UoS-7691 (*n* = 3); UoS-7692 (*n* = 3); UoS-8052 (*n* = 4); UoS-9645 (*n* = 9).

The MET channel is a route of aminoglycoside entry into sensory hair cells (25–28), and various MET channel blockers provide protection against aminoglycoside toxicity (20, 24, 34–36). To assess channel block as a protective mechanism, interactions between the MET channel and the 8 compounds identified from the initial screen were investigated. MET currents elicited by hair-bundle stimulation were recorded from OHCs at membrane potentials ranging from –164 mV to +96 mV before, during, and after extracellular perfusion with 50 μM of each compound (Figure 5). Examples of these currents are shown for compound UoS-7691, a weak blocker (Figure 5A), and UoS-8052, a stronger blocker (Figure 5C). The block was partially reversible when superfusion with control solution was resumed for UoS-8052 but not for UoS-7691 (Figure 5, B and D). Fractional block curves were generated, and the current was measured during exposure to the compounds relative to the control current recorded before perfusion plotted at each membrane potential (Figure 5E). All 8 compounds interacted with the MET channel, reducing currents across the entire range of membrane potentials to a greater or lesser degree. UoS-3606 and UoS-7692 provided the strongest block, whereas UoS-9645 and UoS-7691 were the weakest MET channel blockers.

UoS-7692 thus provided complete protection of OHCs at 50 μM when tested against 5 μM gentamicin and was not toxic when tested alone at 100 μM. UoS-7692 also blocked the MET channel, but it did not block *I*_K,neo_ and is therefore unlikely to reduce drug entry indirectly. On the basis of these properties, it was selected for further detailed characterization to determine (a) dose-response relationships, (b) the ability to protect against different aminoglycoside antibiotics, (c) the impact on the bactericidal activity of gentamicin, (d) a likely mechanism of action, and (e) if it would provide protection of the cochlea in vivo.

### Dose-response relationship for protection of mouse OHCs by UoS-7692.

**Figure 6:**
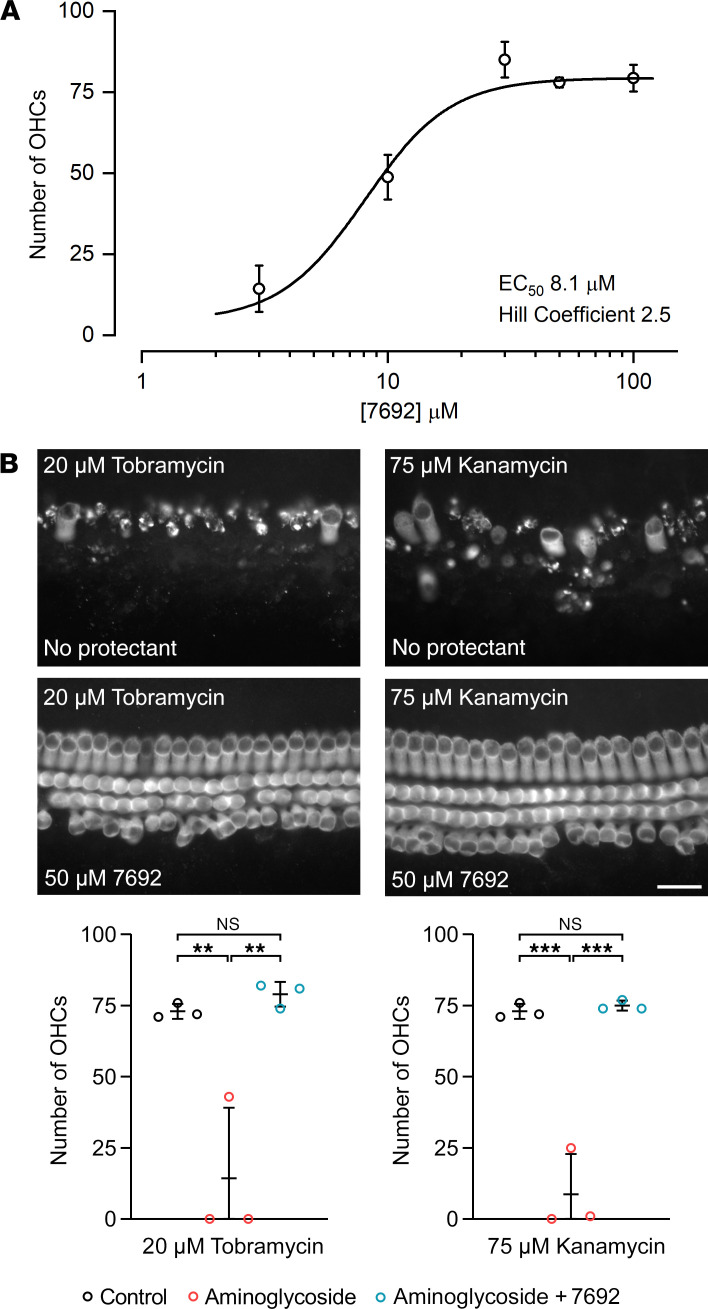
Dose-response function for gentamicin protection with UoS-7692 and protection from tobramycin and kanamycin by UoS-7692. (**A**) Dose-response graph showing average number of OHCs in mid-basal ROI from cultures exposed to LSM with 5 μM gentamicin and 3–100 μM UoS-7692 for 48 hours. Graph fitted with equation 1. EC_50_ value of UoS-7692 is 8.1 μM. Number of cultures: 3 μM (*n* = 3); 10 μM (*n* = 5); 30 μM (*n* = 3); 50 μM (*n* = 6); 100 μM (*n* = 3). Error bars show SEM. (**B**) Micrographs of mid-basal region of cochlear cultures incubated for 48 hours in 20 μM tobramycin (left) or 75 μM kanamycin (right) without (top) or with (bottom) 50 μM UoS-7692. Scale bar: 25 μm. Quantification of OHC survival showed protection from tobramycin- and kanamycin-induced hair-cell death. One-way ANOVA with Tukey’s multiple-comparison test. **P < 0.01; ***P < 0.001. Experiments were performed on 3 independent occasions. Symbols represent data from individual cells. Error bars show SDM.

To determine the EC_50_ for UoS-7692, the concentration at which it provides half-maximal protection from gentamicin damage, cochlear cultures were exposed to 5 μM gentamicin for 48 hours with concentrations of UoS-7692 varying from 3 to 100 μM. Numbers of surviving OHCs in the mid-basal region were counted and a dose-response function was generated (Figure 6A). This function was fit with the equation:

 (Equation 1)
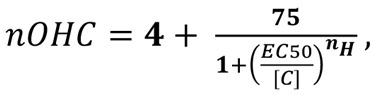


where *nOHC* is OHC count, [C] concentration of protective compound, and *n_H_* the Hill coefficient. The baseline is the average number of OHCs remaining in the mid-basal region of interest (ROI) in cultures exposed to 5 μM gentamicin (4 ± 3 OHCs, *n* = 7), and maximum survival is the average number of OHCs in the same region in control cultures (79 ± 8 OHCs; *n* = 6). The EC_50_ value for UoS-7692 was 8.1 μM, and the Hill coefficient was 2.5.

### UoS-7692 also protects mouse OHCs against kanamycin and tobramycin.

To determine whether UoS-7692 would provide protection against other clinically relevant aminoglycosides, it was tested against tobramycin and kanamycin in mouse cochlear cultures. As shown in Figure 6B, at 50 µM UoS-7692 was found to protect against hair-cell loss induced by either 20 µM tobramycin or 75 µM kanamycin, concentrations of the 2 aminoglycosides that caused a substantial loss of OHCs in the mid-basal coil in the absence of a protectant. UoS-7692 was therefore able to protect hair cells against multiple aminoglycosides.

### UoS-7692 does not compromise the antibacterial activity of gentamicin.

**Figure 7:**
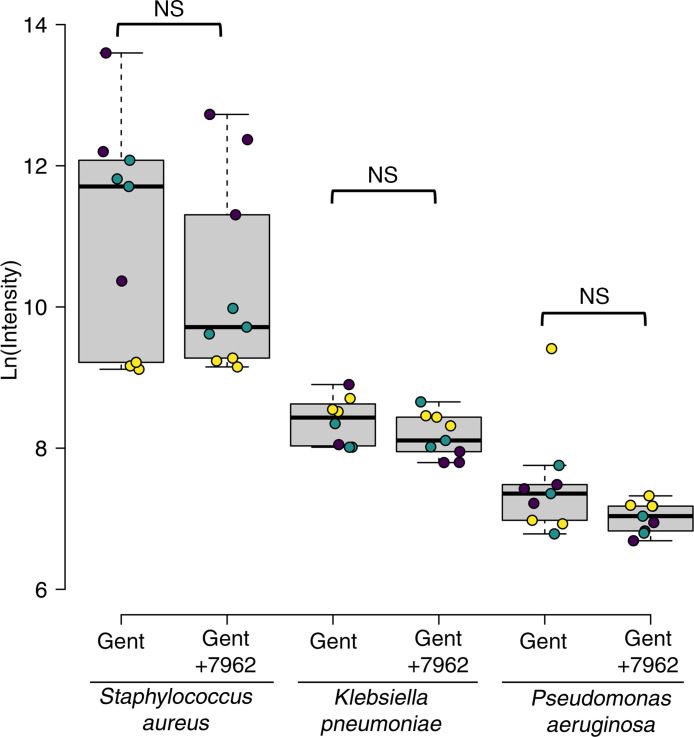
Antimicrobial activity of gentamicin is unaffected by the presence of UoS-7692. Box-and-whisker plots of ATP luminescence as a measure of bacterial viability in *Staphylococcus aureus*, *Klebsiella pneumoniae*, and *Pseudomonas aeruginosa* grown in 2.2 μM gentamicin in the presence or absence of 11 μM UoS-7692. There was no change in the bactericidal activity of gentamicin in the presence of UoS-7692. UoS-7692 was tested with 3 technical replicates and 3 independent biological replicates. Midline = median, boxes = IQR, whiskers = an additional 1.5 × IQR; replicates are shown as open circles with biological replicates grouped by color. *Staphylococcus aureus* (*t* = 1.55, *df* = 14, *P* = 0.143), *Klebsiella pneumoniae* (*t* = 1.44, *df* = 13, *P* = 0.175), and *Pseudomonas aeruginosa* (*t* = 1.71, *df* = 14, *P* = 0.11) using a linear mixed-effects model.

When tested against 3 clinically relevant bacterial pathogens, *Staphylococcus aureus*, *Klebsiella pneumoniae*, and *Pseudomonas aeruginosa*, the bactericidal properties of gentamicin were unaffected in the presence of a 5× higher concentration of UoS-7692 (Figure 7).

### UoS-7692–MET channel interaction reveals potential protective mechanism.

**Figure 8:**
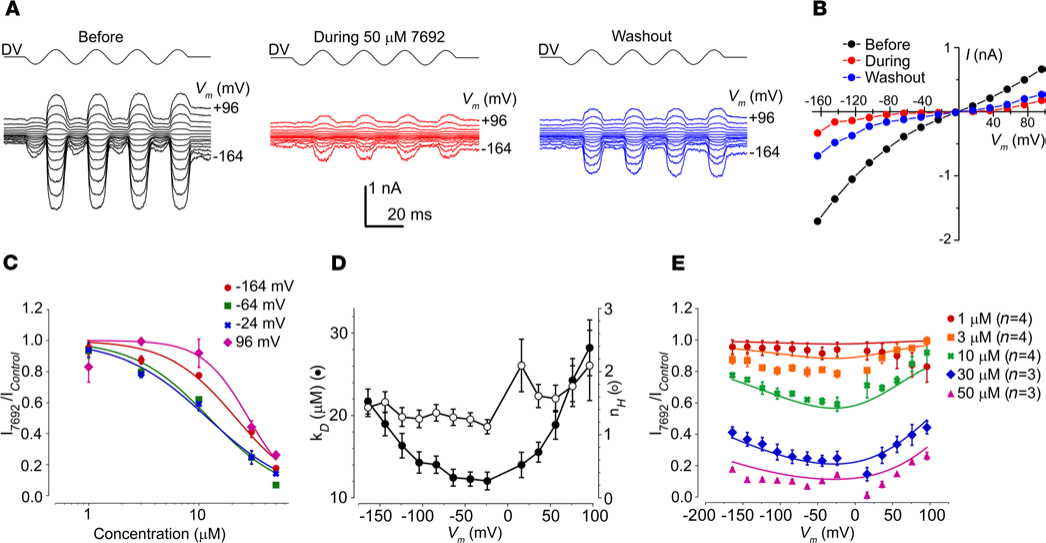
UoS-7692 is a strong MET channel blocker at 50 μM. (**A**) MET currents recorded from an OHC before, during, and after extracellular exposure to 50 μM UoS-7692. Currents elicited by sinusoidal fluid-jet stimulation (DV) and recorded at membrane potentials ranging from –164 to +96 mV. (**B**) Current-voltage curves before, during, and after extracellular exposure to 50 μM UoS-7692. Cell capacitance was 7.4 pF. (**C**) Average dose-response curves for MET channel block by UoS-7692, fitted to equation 2. For clarity, data for only 4 selected membrane potentials are shown. (**D**) *K_D_* and Hill coefficient from dose-response curves fitted to data recorded at each membrane potential. (**E**) Average fractional block curves showing current during UoS-7692 superfusion relative to control current at each membrane potential. Curves are fits to a 2-barrier 1 binding-site model (equation 3). Fit parameters: *ΔE* 0.0 kT (95% CI –1.2 to 1.2 kT); *E_b_* –17.0 kT (95% CI –18.3 kT to –15.7 kT); *δ_b_* 0.69 (95% CI 0.51 to 0.87); *z* 0.73 (95% CI +0.72 to +0.74); *n_H_* 1.45 (95% CI 1.34 to 1.56). Number of OHCs in **B**–**D**: 1 μM (*n* = 4); 3 μM (*n* = 4); 10 μM (*n* = 4); 30 μM (*n* = 3); 50 μM (*n* = 3); error bars show SEM.

To characterize the interaction between UoS-7692 and the MET channel, MET currents were recorded at different membrane potentials before, during, and after extracellular perfusion with 50 μM UoS-7692 (Figure 8A). The corresponding current-voltage curves (Figure 8B) showed some recovery of the current after washout with control solution, indicating that the block was reversible. The recovery was not as immediate as that previously reported for other MET channel blockers, including d-tubocurarine and berbamine (34), and took time to develop, suggesting UoS-7692 does not readily dissociate from the channel.

To determine how the block varied with concentration, currents were recorded during extracellular exposure to concentrations of UoS-7692 between 1 and 50 μM. Dose-response curves derived from the currents measured at each membrane potential (selected membrane potential levels shown in Figure 8C) revealed an increase in the block with increasing concentrations of UoS-7692. The half-blocking concentrations (*K_D_*) and Hill coefficients determined from each dose-response curve are shown in Figure 8D. The *K_D_* values at extreme hyperpolarized and depolarized potentials were 21.5 μM and 28.2 μM, respectively, dropping to 12.0 μM at –24 mV, reflecting a stronger block at intermediate potentials (Figure 8D). The Hill coefficients ranged from 1.1 to 2.1 (Figure 8D), suggesting that more than one molecule of UoS-7692 binds to the channel, showing positive cooperativity (37). At the resting potential of early postnatal mouse OHCs (–55 to –60 mV; refs. 21, 33, 38), the *K_D_* was 12.4 μM (Figure 8C), a value close to that found for the EC_50_ of UoS-7692 in the hair-cell protection assay (8.1 μM, Figure 6A).

Average fractional block data confirmed the increase in the level of the block with increasing concentration of UoS-7692 (Figure 8E). At the higher concentrations (10–50 μM), there was a release of the block at both the extreme depolarized and hyperpolarized potentials, with release at hyperpolarized potentials indicative of a permeant blocker. The fractional block data were fitted with Equation 4, described in the Methods. From the fits, it can be deduced that UoS-7692 blocks maximally at a membrane potential of –3 mV, is positively charged (valence +0.73), and that it binds (binding energy –17.0 *kT*) to a site with a relative electrical distance, as a fraction of the transmembrane electrical field, of 0.69 from the outside of the membrane. The binding site for UoS-7692 is close to that for dihydrostreptomycin (relative electrical distance from the outside = 0.79), but it binds much more strongly (the binding energy for dihydrostreptomycin under similar conditions was –8.27 *kT*).

### UoS-7692 blocks uptake of gentamicin conjugated to Texas red.

**Figure 9:**
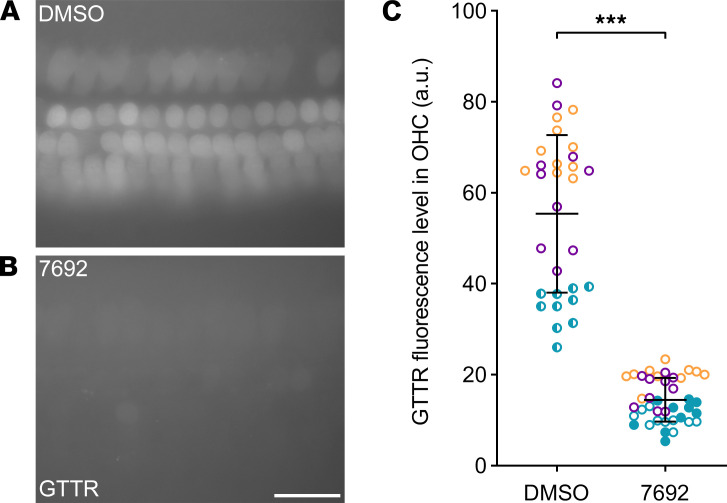
UoS-7692 reduces GTTR loading in cochlear hair cells. Representative micrographs of GTTR fluorescence in cochlear cultures incubated with DMSO (**A**) and UoS-7692 (**B**) prior to the addition of GTTR. Scale bar: 25 μm. (**C**) Comparison of GTTR fluorescence levels in AU by 2-tailed unpaired *t* test shows a reduction in GTTR loading in cultures treated with UoS-7692 (***P < 0.001). Symbols represent individual OHC fluorescence levels (10 cells from 3 control and 4 UoS-7692–treated cultures: different colors indicate the 3 independent experimental repeats). Error bars show SDM.

Although the decrease in MET current size in the presence of UoS-7692 showed that this compound blocked the MET channel, it is important to determine whether UoS-7692 decreases aminoglycoside loading. To assess this, cultures were incubated with 100 μM UoS-7692 for 5 minutes and gentamicin conjugated to Texas red (GTTR) was added for a further 10 minutes before washout and imaging. Incubation with GTTR in the presence of DMSO resulted in strong labeling of the OHCs (Figure 9A). After incubation with GTTR in the presence of UoS-7692 (Figure 9B), the average level of GTTR loading was reduced significantly (Figure 9C).

### UoS-7692 preserves larval movement in neomycin-treated zebrafish.

**Figure 10:**
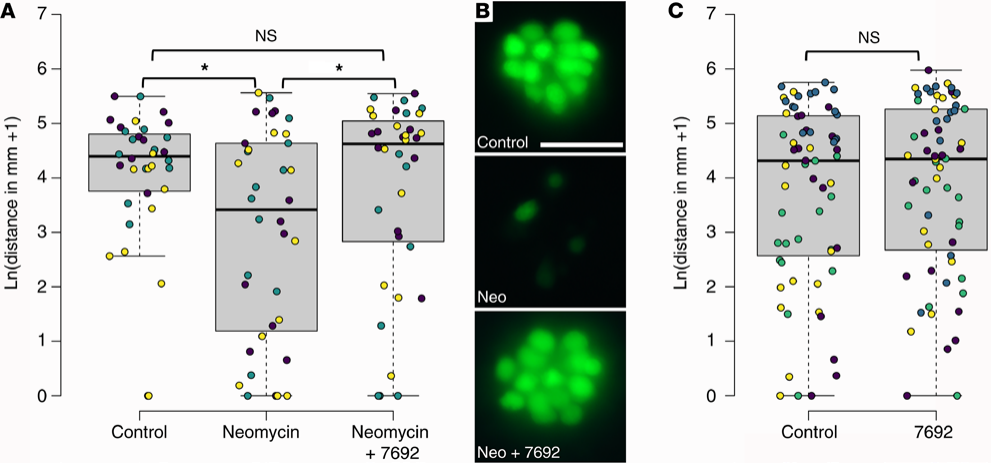
UoS-7692 preserves larval movement in neomycin-treated zebrafish. (**A**) Box-and-whisker plots showing movement of 5 dpf zebrafish larvae after treatment with E3 medium, 6.25 μM neomycin, or 25 μM UoS-7692 and 6.25 μM neomycin, followed by washout. Circles show individual larvae with independent experiments grouped by color (*n* = 3). (**B**) Images of example neuromasts labeled with YO-PRO-1 after treatment with E3 medium (top), 6.25 μM neomycin (middle), or 25 M UoS-7692 and 6.25 μM neomycin (bottom) after behavioral testing. Scale bar: 20 μm. (**C**) Box-and-whisker plots showing movement of 5 dpf zebrafish after treatment with either E3 medium or 25 μM UoS-7692 alone followed by washout. Circles show individual larvae with independent experiments grouped by color (*n* = 4). Black line shows median, gray boxes span the IQR, and whiskers extend over points within additional 1.5 × IQR. Neomycin compared with control larvae *P* = 0.029, neomycin and UoS-7692 compared with control larvae *P* = 0.3947, neomycin and UoS-7692 compared with neomycin alone larvae *P* = 0.0153 (Dunn’s post hoc tests). UoS-7692 alone compared with control larvae *t* = 0.552, *df* = 126, *P* = 0.58 using a 2-sample *t* test. *P < 0.05.

Zebrafish are a powerful in vivo model for assessing the protective potential of compounds, and although many studies have sought to identify compounds that prevent lateral-line hair-cell loss after aminoglycoside exposure, none have determined whether the continued presence of hair cells correlates with preserved behavioral responses. When treated with 6.25 μM neomycin for 1 hour, 5 dpf larvae showed reduced movement after washout compared with control larvae (Figure 10A). After cotreating larvae with 6.25 μM neomycin in the presence of 25 μM UoS-7692, larval movement after washout was not significantly different from that in untreated controls but was significantly greater than movement in larvae treated with neomycin alone (Figure 10A). Labeling with YO-PRO-1 confirmed that hair cells were absent in larvae treated with neomycin alone but still present in hair cells cotreated with neomycin and UoS-7692 (Figure 10B). Treatment of larvae with 25 μM of UoS-7692 alone did not significantly reduce larval movement after washout relative to control larvae (Figure 10C).

### UoS-7692 protects mouse cochlear hair cells from furosemide/kanamycin exposure in vivo.

**Figure 11:**
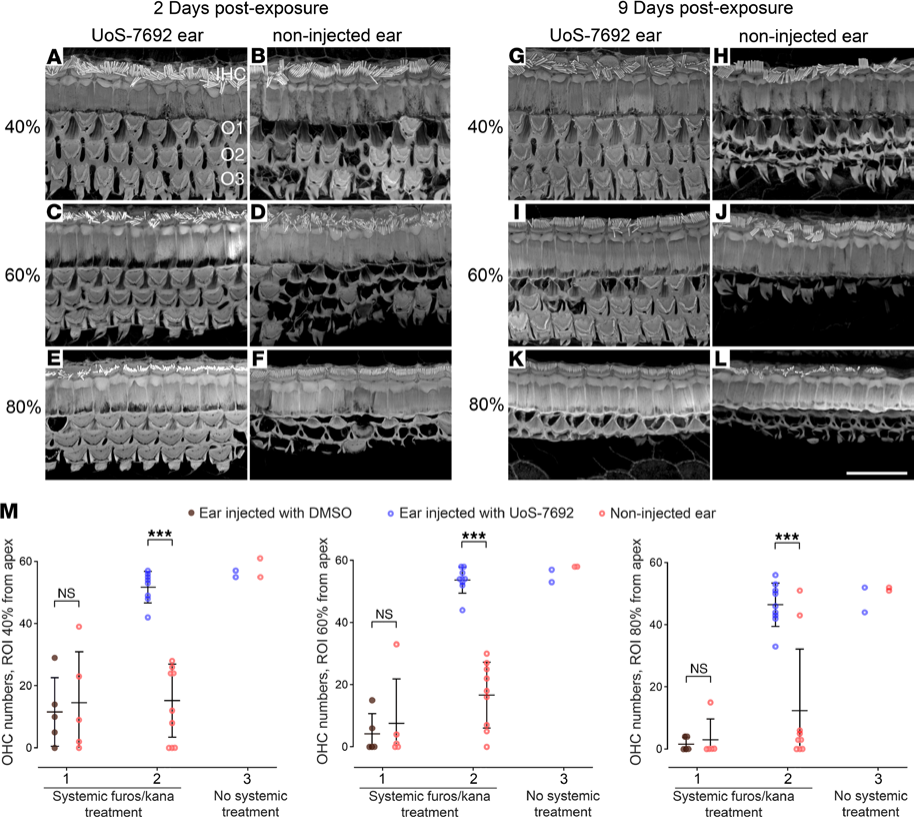
Transtympanic injection of UoS-7692 protects OHCs from systemic kanamycin treatment. (**A**–**L**) Assessment of OHC-bundle morphology with phalloidin staining in regions 40% (**A**, **B**, **G**, and **H**), 60% (**C**–**D**, **I**, and **J**), and 80% (**E**, **F**, **K**, and **L**) from the cochlear apex in mice receiving transtympanic injection of UoS-7692 in 1 ear (**A**, **C**, **E**, **G**, **I**, and **K**) and no injection in the opposite ear (**B**, **D**, **F**, **H**, **J**, and **L**), followed by systemic furosemide/kanamycin. Mice were maintained for either 2 (**A**–**F**) or 9 (**G**–**L**) days. In UoS-7692–treated ears after 2 days, there was minimal or no OHC loss in all regions; in untreated ears, OHC loss was extensive, particularly in the 60% and 80% regions. In UoS-7692–treated ears after 9 days, some OHC loss was observed in the 60% region (**I**) and total loss seen in the 80% region (**K**); in untreated ears, there was total loss of OHCs in all regions (**H**, **J**, and **L**). Scale bar: 20 μm. (**M**) Graphs comparing OHC numbers in cochleae of noninjected (red symbols) and injected (brown and blue symbols) ears of mice 2 days after unilateral transtympanic application of 5% DMSO (brown symbols) or 50 mM UoS-7692 in 5% DMSO (blue symbols), followed by systemic furosemide/kanamycin (groups 1 and 2) or no treatment (group 3). Numbers of mice: group 1 (*n* = 5), group 2 (*n* = 9), group 3 (*n* = 2). OHCs were counted in 140 μm length regions located 40%, 60%, and 80% from the cochlear apex. UoS-7692 provided significant protection in all 3 regions (2-way ANOVA with matched pairs design between cochleae from the same animal, with Šidák’s multiple-comparison test between injected and noninjected ears. ***P < 0.001). Scale bar: 20 μm. Error bars show SDM.

Super-resolution microscopy and quantification of the numbers of OHCs were used to determine whether transtympanic delivery of UoS-7692 provides protection in vivo from i.p. injection of a loop diuretic (furosemide) followed by aminoglycoside (kanamycin), a procedure reported to cause rapid, reliable, and extensive loss of hair cells (39). Confocal images collected at defined points along the cochlea from mice 2 days after furosemide/kanamycin exposure revealed that UoS-7692–injected ears typically retained a full complement of OHCs in regions 40% and 60% from the apical, low-frequency end (Figure 11, A and C), whereas substantial OHC loss was seen in ears that did not receive UoS-7692 (Figure 11, B and D). In the 80% region, the OHC complement was near normal in the UoS-7692–injected ears (Figure 11E), whereas OHC loss was extensive in ears that did not receive UoS-7692 (Figure 11F). Nine days after furosemide/kanamycin exposure, OHC numbers in the 20% (not shown) and 40% regions (Figure 11G) were normal in ears that had received UoS-7692. Some loss, however, was observed in the 60% region (Figure 11I), and a total loss of OHCs was seen in the more basal, 80% region (Figure 11K). In noninjected ears of mice exposed to furosemide/kanamycin alone, there was total loss of OHCs in all regions (20% [data not shown], 40%, 60%, and 80% from apical end) after 9 days (Figure 11, H, J, and L). In surviving hair cells, hair bundles were typically intact and of normal appearance. Staining with prestin antibodies revealed the cell bodies of surviving OHCs also appeared normal (Supplemental Figure 2). Hair-cell complement and hair-bundle morphology were normal in ears that received either UoS-7692 or 5% DMSO without subsequent systemic furosemide/kanamycin treatment and in cochleae from animals that received furosemide alone (data not shown).

Quantification of OHC numbers (Figure 11M) indicated that there was significant loss of OHCs in all regions 2 days after furosemide/kanamycin exposure and that this was not alleviated by transtympanic injection (TTI) of 5% DMSO alone (Figure 11M, group 1). However, TTI of UoS-7692 (in 5% DMSO) resulted in significant increases in OHC numbers surviving furosemide/kanamycin exposure relative to those in noninjected ears in all regions (Figure 11M, group 2).

### UoS-7692 partially preserves hearing in mice after furosemide/kanamycin exposure.

**Figure 12:**
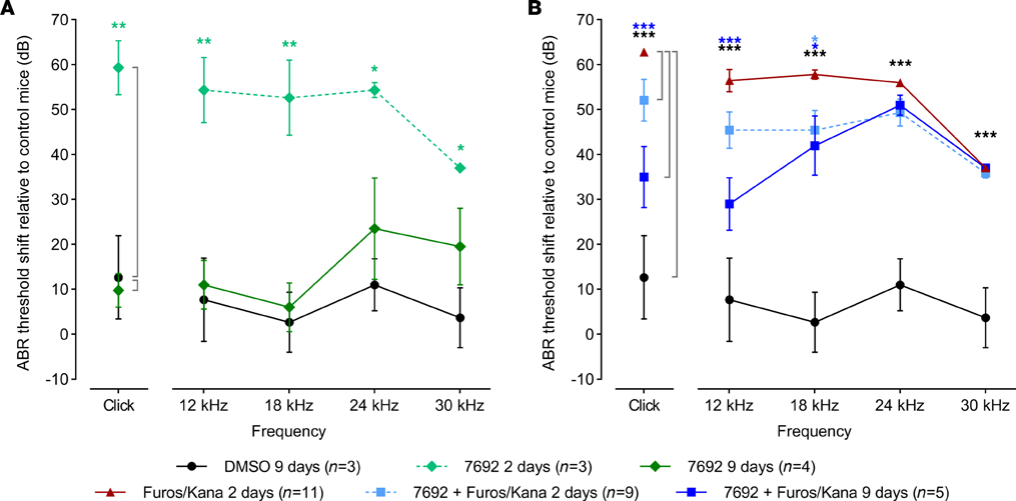
Transtympanic injection of UoS-7692 reduces kanamycin-induced hearing loss in mice. (**A**) ABR thresholds shifts from baseline (0 dB) measured 9 days after bilateral trans-tympanic injection (BTTI) of 5% DMSO (black circles, black line), 2 days after BTTI of 50 mM UoS-7692 (light-green diamonds, dashed line), and 9 days after BTTI of 50 mM UoS-7692 (dark-green diamonds, solid line). UoS-7692 induced significant temporary threshold shifts at 2 days, which were resolved by 9 days (1-way ANOVAs for click and individual pure-tone frequencies with Dunnett’s multiple-comparison test between UoS-7692 at 2 and 9 days and DMSO at 9 days). Significance levels for differences between UoS-7692 at 2 days and DMSO at 9 days are indicated by light-green asterisks. (**B**) ABR threshold shifts measured 9 days after BTTI of 5% DMSO (black circles), 2 days (light-blue squares, dashed line), 9 days (dark-blue squares, solid line) after 50 mM UoS-7692 BTTI followed by furosemide/kanamycin exposure, and 2 days after furosemide/kanamycin exposure without BTTI (red triangles). BTTI of UoS-7692 significantly reduced threshold shifts 9 days after furosemide/kanamycin exposure for clicks and pure-tone frequencies of 12 and 18 kHz (1-way ANOVAs for click and individual pure-tone frequencies with Dunnett’s multiple-comparison test between the 2-day furosemide/kanamycin condition and the other 3 conditions). Significance levels for differences between Furos/Kana at 2 days and UoS-7692 + Furos/Kana at 2 and 9 days are indicated by light- and dark-blue asterisks, respectively; significance levels for differences between Furos/Kana at 2 days and DMSO at 9 days are indicated by black asterisks. *P < 0.05; **P < 0.01; ***P < 0.001. *N* numbers are shown in the key. Error bars show SEM.

Auditory brainstem response (ABR) thresholds for click and pure-tone stimuli were also measured to determine the extent to which transtympanic delivery of UoS-7692 would protect hearing in mice treated with furosemide/kanamycin. ABRs recorded from mice 9 days after TTI of 5% DMSO in both ears without subsequent furosemide/kanamycin exposure revealed a small, nonsignificant elevation in ABR thresholds (Figure 12A, black line/symbol), relative to those of control mice. Mice that received UoS-7692 in both ears, but were not exposed to furosemide/kanamycin, had threshold shifts greater than 50 dB after 2 days (Figure 12A, dashed light-green line/diamonds). However, 9 days after TTI of UoS-7692 alone, these threshold shifts were much reduced (Figure 12A, dark-green line/diamonds) and not significantly different from those following TTI of DMSO alone. UoS-7692 alone therefore caused a temporary threshold shift, as would be expected for a MET channel blocker and probably due to a slow clearance rate from the inner ear.

Two days after exposure to furosemide/kanamycin alone, and as expected from the observed loss of OHCs (see Figure 11, B, D, F, and M), auditory thresholds were significantly increased relative to those in controls that were transtympanically injected with DMSO but not treated with furosemide/kanamycin, both for clicks and at all pure-tone frequencies tested (Figure 12B, red symbols). With prior TTI of UoS-7692, auditory thresholds were also increased 2 days after furosemide/kanamycin exposure (Figure 12, light-blue line/squares), even though OHC numbers were normal (see Figure 11, A, C, E, and M). After 9 days, however, although minimal improvement was seen at 24 and 30 kHz, mice that received transtympanic UoS-7692 followed by furosemide/kanamycin (Figure 12B, dark-blue line/squares) had significantly improved thresholds at 12 and 18 kHz, relative to those measured 2 days after furosemide/kanamycin alone (Figure 12B, red line/triangles). Improvements of approximately 30 dB were observed for clicks and 12 kHz tones. Therefore, although transtympanic application of UoS-7692 caused a temporary loss of hearing, it largely prevented hair-cell loss throughout the cochlea and reduced, at lower frequencies, deafness caused by loop diuretic/aminoglycoside treatment.

## Discussion

The results describe a screen of 10,240 compounds identifying 8 hits that provide protection against gentamicin-induced hair-cell loss in mouse cochlear cultures without causing overt hair-bundle damage. After further selection using stringent criteria, UoS-7692 was characterized in depth. UoS-7692 significantly reduced GTTR accumulation in mouse OHCs in vitro, did not compromise the bactericidal properties of gentamicin, and protected zebrafish against the behavioral consequences of neomycin-induced hair-cell loss. Furthermore, UoS-7692 protected hair cells and partially preserved hearing in mice after exposure to a combination of an aminoglycoside and a loop diuretic, a procedure that eliminates almost all cochlear OHCs within 48 hours (39).

**Figure 13:**
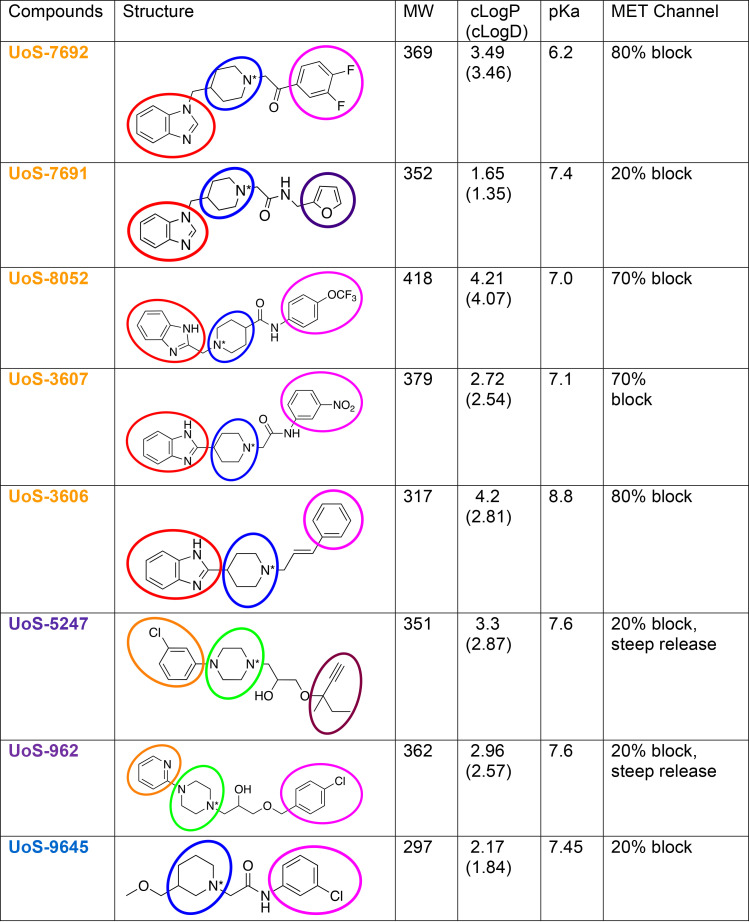
Structures and properties of the Diversity Set hits. Table illustrating structures and properties of the 8 compounds from the Life Chemicals Diversity Set that protect mouse OHCs from exposure to gentamicin in vitro. Names of compounds in the first column are colored according to dendrogram group shown in Figure 1. The molecules and their substructure classification are based on a working model that assumes likely commonality in the mode of binding to the channel. Properties shown are molecular weight (MW), lipophilicity (cLogP and cLogD), calculated acid dissociation constant (p*K*a), and fractional (%) block of MET current by 50 μM of compound at a holding potential of –164 mV. The basic *N* is indicated with an asterisk. Moieties outlined are piperidine (blue), piperazine (green), benzimidazole (red), metachlorophenyl, pyridine (orange for UoS-5247 and UoS-962, respectively), and furan (purple). Lipophilic aromatic moiety for UoS-7692, UoS-8052, UoS-3607, UoS-3606, UoS-962, and UoS-9645 is outlined in magenta and lipophilic side chain of UoS-5247 is outlined in maroon.

Despite the chemical diversity of the library, all of the initial hits were found to interact to varying degrees with the MET channel, an established entry route for aminoglycosides into hair cells (25–28). To our knowledge, none of these 8 hits have been reported yet to have any biological effect against other targets nor are similar to ORC-13661. The structure of these 8 hits is shown in Figure 13 with various moieties outlined in different colors. With the exception of UoS-9645 and UoS-5247, these compounds share 3 main parts, which include 2 aromatic moieties linked together by a cyclic amine; the latter is either piperidine (blue for UoS-7692, UoS-7691, UoS-8052, UoS-3607, UoS-3606, and UoS-9645) or piperazine (green for UoS-5247 and UoS-962). As previously reported (21, 34, 36), the presence of a basic amine or quaternary nitrogen (i.e., protonated at plasma pH) is required for the interaction with the MET channel. One of the aromatic moieties, benzimidazole, is recurrent for 5 compounds (red for UoS-7692, UoS-7691, UoS-8052, UoS-3607, and UoS-3606) but is linked differently with regard to position in the benzimidazole ring and to the spacer between the benzimidazole and the piperidine ring. Compounds UoS-5247 and UoS-962 have, instead, a monocyclic aromatic ring (orange), meta-chlorophenyl and pyridine, respectively. The second aromatic moiety, despite being variable, is generally lipophilic (magenta); the exception is UoS-7691, which bears a furan ring (purple). A lipophilic moiety is also shared by UoS-5247, which, despite lack of a second aromatic system, has a lipophilic side chain (maroon). UoS-9645 has the piperidine ring (blue) and a lipophilic chlorophenyl ring (magenta), but it lacks the second aromatic moiety.

The effects of the 8 initial hits on OHC MET currents showed that the compounds bearing a benzimidazole moiety interacted more strongly with the MET channel, with compounds UoS-7692, UoS-8052, UoS-3607, and UoS-3606 providing a more than 70% block of MET current at 50 μM. The exception was UoS-7691, which only provided a 20% block. UoS-7691 has the least lipophilic moiety (a furan ring, purple), compared with the more lipophilic rings of other compounds (magenta), and this may account for its reduced efficacy. The apparent requirement of a lipophilic aromatic moiety for enhanced block is of interest given recent findings indicating the ion conduction pathway of the MET channel subunit, TMC1, may be a groove that abuts the lipid bilayer (40, 41). Lipophilicity may therefore facilitate interactions of the compounds with negatively charged aminoglycoside binding sites lying deep in the conduction path.

Previous studies (21, 34, 36) have established the necessity of either a fixed positive charge or a pH-dependent positively charged moiety, usually a nitrogen with a p*K*a ^3^7.5. The most effective channel blockers were UoS-3606 and UoS-7692, both of which, at 50 μM, provided a block of the MET current of approximately 80%. UoS-3606 had the highest calculated p*K*a (8.8), and more than 90% of molecules are expected to be positively charged at plasma pH (7.4). With a calculated p*K*a of 6.2, UoS-7692 is predicted to be only partially protonated at physiological pH, and it is less easy to interpret how it interacts with the MET channel. The combination of substituents (benzimidazole and difluorophenyl) may, however, favor a stronger interaction with the MET channel and thus contribute to the strong block observed with UoS-7692. Similarly, the 70% block provided by UoS-8052 and UoS-3607, each of which are partially protonated (~50% at pH 7.4), suggests benzimidazole and a lipophilic aromatic moiety are both needed for a strong interaction with and block of the MET channel. Finally, the steep release of the MET current block seen with UoS-962 and UoS-5247 at hyper- and depolarized potentials, along with the very weak block of the MET current observed with UoS-9645, indicate the benzimidazole moiety favors interactions with the MET channel.

The model used (27, 42) to fit the MET channel fractional block data provides further insight into the mechanism whereby UoS-7692 is likely to be providing protection. Despite predictions from the p*K*a, the fits suggest UoS-7692 has a modest positive charge of +0.73. Furthermore, the binding site for UoS-7692 is predicted to be deep inside the channel at a relative electrical distance of 0.69, a position very similar to that of the aminoglycoside binding site. The model also predicts UoS-7692 binds very tightly to the MET channel, more strongly than the aminoglycosides, with a high binding energy comparable to the otoprotectant carvedilol and its derivative compound 13 (36). Overall, these findings suggest UoS-7692 is competing directly with aminoglycosides at a binding site located in the ion conduction pathway of the MET channel, a suggestion supported by its ability to block GTTR loading. However, UoS-7692 and the other hit compounds all behave as permeant blockers of the MET channel and may therefore operate as protectants, alternatively or additionally, at intracellular sites downstream of hair-cell entry.

The protectants discovered in this study add considerably to those that have been identified previously and are also known to interact with the MET channel, such as amiloride, benzamil, berbamine, carvedilol, d-tubocurarine, hexamethyleneamiloride, ORC-13661, and quinine (20, 25, 34, 36, 43–47). Although the MET channel may therefore be a good target for informing the design of an otoprotectant that could be coadministered with aminoglycosides to prevent hair-cell death, this study raises the question of whether compounds that are likely to block the MET channel transiently are a realistic and clinically acceptable option for providing protection against aminoglycosides. Despite this concern, a recent study with ORC-13661 in rats indicates that a systemically applied MET channel blocker can provide protection against aminoglycosides without causing auditory threshold shifts during treatment (24). A likely explanation, based on the observation that patients only experience deafness after, and not during, treatment with aminoglycosides, is that cochlear hair cells accumulate aminoglycosides at a low rate from scala media, an extracellular compartment in which the aminoglycoside concentration may never be very high (31, 48). If so, relatively low concentrations of MET channel–blocking otoprotectants may suffice to compete for entry and reduce aminoglycoside accumulation in hair cells to levels below those required to instigate cell death. Once optimized for systemic delivery, and if tested in a longer-term model with repeated systemic application of aminoglycoside, a compound based on UoS-7692 may also protect hair cells in vivo at a much lower dose and without causing a temporary shift in auditory thresholds.

In conclusion, this study led to the identification of 8 protective compounds, all of which are blockers of the MET channel. As such, it provides further confirmation that this channel is a viable target for the design of otoprotectants. The structures and properties of these compounds, some of which are quite closely related, together with those of MET channel–blocking protectants already known, provide a good starting point for the development of lead compounds that could alleviate the unwanted, off-target effects of the otherwise clinically useful aminoglycoside antibiotics.

## Methods

### Chemical library

Compounds tested were from the Life Chemicals Diversity Set; 10,240 compounds were supplied predissolved and preplated at 10 mM in DMSO. Molecules effective in the initial screens were confirmed by testing new 10 mM stocks made from solid samples that were obtained from Life Chemicals and analyzed in house to confirm the structure and purity of each compound, diluted in DMSO (MilliporeSigma, G3632), and divided into single-use aliquots stored at –80°C. Physicochemical properties of compounds were calculated using MarvinSketch 20.11 by ChemAxon (https://www.chemaxon.com).

### Zebrafish neomycin and gentamicin protection assays

Zebrafish embryos were obtained from sibling crosses of adult *nacre* (*mifta^–/–^*) fish (49) maintained at the University of Sussex or University of Sheffield. The rationale for using this line has been described previously (21). Larvae were maintained and all assays were performed in E3 medium (5 mM NaCl, 0.17 mM KCl, 0.33 mM CaCl_2_, 0.33 mM MgSO_4_). In brief, larvae at 4 days dpf were exposed to YO-PRO-1 (Invitrogen, Y3603) to selectively prelabel the hair cells (50) and dispensed 3 to 4 per well into 96-well microtiter plates (Greiner). Larvae were incubated at 28°C for 1 hour in 6.25 μM neomycin sulphate (MilliporeSigma, N1876) in the presence of 25 μM test compound or for 6 hours in 10 μM gentamicin sulphate (MilliporeSigma, G3632) in the presence of 50 μM test compound. The doses of aminoglycosides and the exposure times used have been previously shown to kill 75%–100% of hair cells in the trunk neuromasts (21, 34). Each row of the plate included an aminoglycoside-only control and an untreated control to confirm death or health of hair cells. After washout of compounds and aminoglycosides, plates were screened using a 16× 0.40 NA objective and 15× eyepieces on a Zeiss IM-35 inverted microscope. Posterior lateral-line (trunk) neuromasts 3 to 9 were viewed and qualitatively assessed as described in Supplemental Methods. Images of neuromast 4 were recorded with a 40× 0.75 NA objective using a Nikon D5000 camera.

### Mouse cochlear culture preparation

Mouse cochlear cultures were prepared from wild-type CD-1 mice of either sex as previously described (51). In brief, P2 pups were killed by cervical dislocation in accordance with UK Home Office guidelines, and heads surface-sterilized in 80% ethanol. Cochleae were removed and placed in HBSS (Gibco, Thermo Fisher Scientific, 14025050) buffered with 10 mM HEPES (HBHBSS; MilliporeSigma, H0887) for further dissection. Organs of Corti were plated onto collagen-coated (Corning, 354236) coverslips in 93% DMEM-F12, 7% FBS, and 10 μg/mL ampicillin (a non-ototoxic penicillin-type antibiotic), sealed into Maximow slide assemblies, and left to grow and adhere to the collagen for 24 hours at 37°C.

### Mouse cochlear culture protection assay

After 24 hours of incubation, coverslips with adherent cochleae were placed in 35 mm Petri dishes (Greiner Bio-One, 627161), and 1 mL LSM (98.8% DMEM/F12, 1.2% FBS) containing either (a) vehicle (0.5% DMSO), 5 μM gentamicin in the presence of 0.5% DMSO, or 5 μM gentamicin with varying concentrations of compound, or (b) either 100 µM of protectant alone or 1% DMSO. Cultures were then incubated for 48 hours at 37°C. UoS-7692 was also tested at 50 μM against 20 μM tobramycin (MilliporeSigma, T1783) or 75 μM kanamycin (MilliporeSigma, K1377) as described above. After 48 hours, cultures were washed in PBS, fixed at room temperature for 1 hour in 3.7% formaldehyde (v/v) (MilliporeSigma, F1635) in 0.1 M sodium phosphate pH 7.4, and then incubated at 4°C overnight in PBS containing 10% horse serum and 0.1% Triton X-100 with 1:200 Texas red phalloidin (Invitrogen, Thermo Fisher Scientific, T7471) and 1:1000 rabbit anti-myosin VIIa (Proteus Bio-Sciences, 25-6790), followed by 4 hours at room temperature in 1:500 Alexa Fluor 488 goat anti-rabbit secondary antibody (Invitrogen, Thermo Fisher Scientific, A-11034). Cultures were mounted in Vectashield (Vector Laboratories, H-1000) and imaged using a 40× 0.75 NA objective on a Zeiss Axioplan2 upright microscope with a Spot RT Slider camera. Images were obtained from multiple focal planes and merged using Adobe Photoshop Creative Cloud. Numbers of hair cells were counted in a 221 μm (1200 pixel) mid-basal region, approximately 20% along the length of the cochlea from the basal end. OHCs were counted if the hair-bundle was present and if the cell soma was not severely swollen, condensed, or fragmented.

### Chemical screen data visualization

Life Chemicals Diversity compounds’ chemical structures were standardized using the Molecular Operating Environment wash procedure of the Chemical Computing Group Inc. accessed through the Konstanz Information Miner (52). Molecules were analyzed using RDKit (http://www.rdkit.org) in Python (https://www.python.org). Compound similarity was calculated using the Tanimoto coefficient (53) of the Morgan fingerprints of radius 2, which are equivalent to ECFP4 (54), using the scikit-learn library (55). A dendrogram was obtained based on all pairwise similarity values between all compounds using the SciPy library (http://www.scipy.org). The dendrogram and the polar scatterplot were visualized using the matplotlib library (56).

### Electrophysiology

MET currents and basolateral potassium currents were recorded and analyzed as previously detailed (34). In brief, whole-cell currents were recorded from OHCs in organotypic cultures that had been prepared from P2 CD-1 mice and maintained in vitro for 1 to 2 days. MET currents were recorded from basal-coil OHCs (to match the location of hair cells in the protection assays) at membrane potentials ranging from –164 mV to +96 mV, in the absence or presence of otoprotective compounds (50 μM). MET currents were also recorded during exposure to varying concentrations of UoS-7692 (1–50 μM). Superfusion of all compounds was continued until the block appeared to reach a steady level, before superfusing control solution for as long as possible (up to 10 minutes) to evaluate reversibility of the block. CsCl (137 mM) was included in the intracellular solution to block basolateral potassium channels. Currents were elicited by stimulating hair bundles using a fluid jet from a pipette (tip diameter 8–10 μm) driven by a piezoelectric disc (57). Mechanical stimuli (filtered at 1.0 kHz, 8-pole Bessel) were 45 Hz sinusoids with driver voltage amplitudes of ±40 V. Dose-response curves of MET current block by UoS-7692 were determined for all membrane potentials tested and fitted with the equation:

(Equation 2)
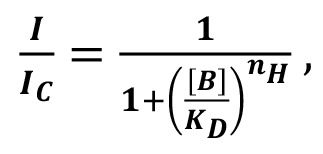


where *I_C_* is control MET current, [*B*] is concentration of the blocking compound, *K_D_* is half-blocking concentration, and *n_H_* is the Hill coefficient.

Basolateral currents were recorded at membrane potentials ranging from –154 to +46 mV in the absence or presence of 50 μM of the otoprotective compounds, again taking care to reach steady-state block and maximizing time for the block to reverse. CsCl was substituted by KCl (131 mM) in the intracellular solution. Currents were acquired using pClamp (Molecular Devices) software.

During recordings, series resistance compensation (~70%) was applied to limit the voltage drop across this resistance. Following compensation, the average residual series resistance was calculated to be 1.69 ± 0.08 MΩ (*n* = 58). MET currents and steady-state basolateral potassium currents reached maximum sizes of 1.43 ± 0.10 nA (*n* = 40) and 1.93 ± 0.11 nA (*n* = 18), respectively, resulting in voltage drops across the residual series resistance of less than 5 mV, sufficiently small not to require correction to quoted voltage values. All experiments were conducted at 20°C–24°C.

### Two-barrier 1 binding-site model of permeant MET channel block

Permeation and block of the MET channel for compound UoS-7692 were quantified by fitting a 2-barrier 1 binding-site model to the fractional block curves as described earlier (27, 34, 42), i.e., similar to that used to describe block of the MET currents by dihydrostreptomycin (33) but modified to allow for Hill coefficients that differ from unity. In plotting the fractional block curves for the specific concentrations ([*B*]) tested, the half-blocking concentration *K_D_* in Equation 2 above becomes voltage dependent:

(Equation 3)
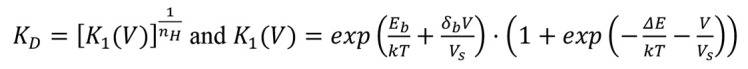


yielding:

(Equation 4)
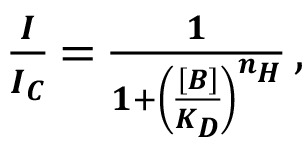


where *E_b_* is the free energy of the binding site, *ΔE* is the height of the intracellular energy barrier minus the height of the extracellular energy barrier, the slope factor: is the ratio of thermal energy (*kT*, i.e., Boltzmann’s constant multiplied by absolute temperature) and effective charge of the blocker molecule (*ze*_0_, i.e., valence multiplied by elementary charge), and *δ_b_* is the relative electrical distance of the binding site across the membrane, measured from the extracellular side. Fits of the fractional block curves were optimized across the range of UoS-7692 concentrations tested using a purpose-written Python 3.0 program.

### Bacterial growth conditions and antimicrobial drug susceptibility assay

Bacterial growth and antimicrobial susceptibility screens were performed as described previously (21). Briefly, *Staphylococcus aureus*, *Klebsiella pneumoniae*, and *Pseudomonas aeruginosa* were passaged twice on Luria-Bertani agar from frozen stocks and cultured in Mueller Hinton Broth at 37°C with shaking at 200 RPM. The minimum inhibitory concentration (MIC) of gentamicin was determined by microbroth dilution method to be 2.2 μM for all 3 strains. To test whether UoS-7692 abrogated the antimicrobial efficacy of gentamicin, bacteria (3–5 × 10^5^ CFU/mL) were exposed to a 1:5 mixture of gentamicin and UoS-7692 (MIC gentamicin [2.2 μM]/UoS-7692) and incubated at 37°C for 16–20 hours. Bacterial ATP was quantified as a measure of viability using the BacTiter-Glo Microbial Cell Viability Assay (Promega) according to the manufacturer’s instructions. The experiment was repeated in triplicate.

### GTTR loading assay

Loading of GTTR (a gift from Peter Steyger, Creighton University School of Medicine, Omaha, Nebraska, USA) was assayed as described previously (21). In brief, 1-day old cochlear cultures prepared from P2 mice were incubated in 0.1% DMSO or 100 μM UoS-7692 in HBHBSS for 5 minutes, and GTTR was added to a final concentration of 0.2 μM for a further 10 minutes. Cultures were washed 3 times with HBHBSS, placed in a viewing chamber with fresh HBHBSS, and GTTR fluorescence was recorded with a 63× 0.90 NA water immersion lens on a Zeiss Axioplan2 upright microscope with a Spot RT Slider camera. Images were collected at a fixed distance from the basal end of the culture 10 minutes after the onset of GTTR washout. Experiments were performed at 22°C to 24°C and on 3 independent occasions. For each culture, intensity values were obtained from 40 × 40 pixel ROIs from 10 consecutive OHCs in a row and 4 background regions. The average of the background fluorescence values was subtracted from each individual OHC value to give the final intensity value for each OHC.

### Measurements of motility in zebrafish larvae

Zebrafish larvae (5 dpf) were treated with E3, E3 containing 6.25 μM neomycin, or E3 containing 25 μM UoS-7692 and 6.25 μM neomycin for 1 hour, washed 3 times with E3 media, and 12 fish from each treatment group were then dispensed into individual wells of a 48-well plate in 1 mL of E3. Larvae were left for 30 minutes at 28°C to acclimatize to the wells before larval movement was tracked in the light using a Noldus Daniovision Observation Chamber over a 30-minute period. After tracking, 3 larvae from each treatment group were labeled with 3 μM of YO-PRO-1 for 15 minutes and assessed on a Zeiss IM35 inverted microscope as described above. Experiments were repeated on 3 independent occasions.

To ensure treatment with compound UoS-7692 alone does not alter the behavior of the larvae, 5 dpf zebrafish were treated with either E3 medium or 25 μM UoS-7692 in E3 for 1 hour. After washing with E3, 16 fish from each treatment group were dispensed into individual wells of a 48-well plate and assayed as described above.

### TTI of UoS-7692 into the mouse middle ear

Compounds were delivered into the middle ear by TTI using a microinjection system (Ultramicropump 3, WPI). The pump was linked by narrow bore tubing to a handheld 33G beveled needle. Estimates suggest that a 2 to 3 order of magnitude drop in concentration of compound occurs between the middle and inner ear as a result of limited permeability of the window membranes (58). UoS-7692 was therefore used at 50 mM (to give an estimated endolymphatic concentration of 50–500 μM), and was diluted in water containing 5% DMSO to improve round-window permeability (58).

Mice were anesthetized with an i.p. injection of 10 μL/g ketamine/xylazine anesthetic (10 mg/mL ketamine hydrochloride, 1 mg/mL xylazine hydrochloride in water), then positioned on their side for viewing of the desired ear canal. Two holes were made in the tympanum with the needle, 1 posterior and 1 anterior to the incus handle, and 4 to 5 μL of compound was delivered through the anterior hole. After TTI, each mouse was maintained on its side for 20 minutes before i.p. injection of 4 μL/g of xylazine recovery agent (atipamezole hydrochloride 0.25 mg/mL, Pfizer). Each mouse was placed in a recovery cage and monitored until active (~25–40 minutes after atipamezole). For injections in both ears, the mouse was turned on its reverse side after 20 minutes, and the process repeated on the second ear.

### Aminoglycoside treatment of TTI mice

Four hours (+/– 1 hour) after TTI, mice were given an i.p. injection of 15 μL/g furosemide (10 mg/mL stock). Kanamycin (100 mM kanamycin A sulphate [MilliporeSigma, K1876] in HBHBSS) was injected 30 minutes later at 5 μL/g (males) or 3 to 4 μL/g (females) by the i.p. route (doses found to consistently induce comparable hearing loss and OHC toxicity), on the opposite side of the body to the furosemide injection. Mice were housed separately, their diet supplemented with wet mash, and monitored twice daily until ABR assessment, either 2 or 9 days later. Saline injections were given if weight dropped by more than 10% of starting value. No mice showed observable ill effects and all regained normal weight within 2 days.

### ABR measurements

ABR methods were carried out following established procedures (59) using Tucker-Davis Technologies equipment. Heart rate, 70 dB click-evoked ABR, click-evoked ABR level series (20–95 dB), and tone-evoked ABRs (20–95 dB) at 12–36 kHz were carried out with free-field stimuli for all mice at either 2 or 9 days after injection (TTI and/or furosemide/kanamycin). When a waveform was not present at 95 dB, the highest level tested, response threshold was recorded as 100 dB for analysis purposes. Threshold shifts were measured relative to average levels required to elicit a detectable response in a cohort of mice (*n* = 5) 2 days after a single injection of furosemide alone (150 μg/g), a procedure that does not cause hearing loss (Supplemental Figure 3).

### Assessment of cochlear morphology

After ABR measurements, mice were overdosed with anesthetic (0.25 mL of pentobarbital; Vetoquinol). The head was removed after death and bisected, the labyrinths extracted, and the round and oval windows were opened. Cochleae were immersion-fixed (3.7% formaldehyde [v/v], 0.1 M sodium phosphate, pH 7.4) at 4°C overnight. Cochleae were washed 3 times in PBS and decalcified in 0.5 M EDTA pH 8.0, for 3–4 days at 4^o^C, with active agitation. Once decalcified, each cochlea was washed in PBS and sliced into 4 pieces, and the organ of Corti was dissected from each piece using iterative trimming as previously described (60). Pieces were preblocked for 1 hour in PBS containing 10% horse serum and 0.1% Triton X-100 and incubated overnight at 4^o^C in 1:1000 goat anti-prestin antibody (Santa Cruz Biotechnology, sc-22692) and 1:2000 Atto-488 phalloidin (MilliporeSigma, 49409) diluted in preblock. After 3 PBS washes, pieces were incubated in Alexa Fluor 586 donkey anti–goat IgG for 4 hours (Thermo Fisher Scientific, A-11057), washed, and mounted under glass coverslips in Vectashield.

Cochleae were photographed at 10× on a Zeiss AxioPlan2 microscope with a Jenoptik Gryphax Arktur camera, and photomontages of cochleae were generated in Adobe Photoshop CC. Using ImageJ (NIH), the entire length of the cochlear coil was measured, and points 20%, 40%, 60%, and 80% from the apex were marked. Super-resolution images of hair cells were captured from these regions using an Airyscan Zeiss 880 LSM and 63× 1.4 NA oil-immersion objective. *Z*-projections were created in Zeiss LSM Browser 4.2 software using the keep maximum transparency function; threshold, ramp, maximum opacity, and brightness were adjusted to prevent the signal from the cuticular plate from obscuring the hair-bundles. Subsequent images were captured in wide field with a 63× 1.4 NA oil-immersion lens on a Zeiss AxioPlan2 microscope as described above to quantify hair-cell survival at intervals 40%, 60%, and 80% from the cochlear apex. Comparable levels of hair-cell loss were seen in both left and right ears in all mice observed that did not receive otoprotectant. Occasionally, mice that received kanamycin treatment did not show hair-cell loss in nonprotected cochleae, and these mice were excluded from the study to avoid false positives.

### Statistics

#### Mouse cochlear culture protection assay.

OHC survival was compared by 1-way ANOVA with Tukey’s multiple-comparison test between control cultures, cultures treated with aminoglycosides alone, and cultures treated with aminoglycosides and test compounds. Statistical analysis was performed using GraphPad Prism version 8.0. *P* values less than 0.05 were considered significant.

#### Electrophysiology.

Where currents recorded from multiple cells were averaged, means ± SEM are quoted and shown in figures. Values for fitting parameters in dose-response and fractional block curves are reported as means with 95% CI.

#### Bacterial growth conditions and antimicrobial drug susceptibility testing.

Assays were analyzed using linear mixed effects models, modeling the multiple variance components in the hierarchical experimental design (61–63). Each assay (*Staphylococcus aureus*, *Klebsiella pneumoniae*, and *Pseudomonas aeruginosa*) was analyzed separately. Natural logarithm-transformed luminescence was used as the response variable. Compound identity (including compound-free control) was fitted as a single categorical explanatory variable, and pairwise contrasts were assessed, comparing each compound with the control. Technical replication, nested within biological variation, was fitted as random effects, with Gaussian errors. Analysis was performed using the R statistical programming environment, version 3.3.1 (64).

#### GTTR loading assay.

Background-corrected fluorescence values in hair cells treated with GTTR, with and without prior incubation with UoS-7692, were analyzed by a 2-tailed *t* test. Statistical analysis was performed using GraphPad Prism version 8.0.

#### Measurements of motility in zebrafish larvae.

The natural logarithm of the distance traveled over a 1-minute sample window was plotted. Since the data set included some distances of 0 mm, data + 1 was used to avoid ln(0) values in the analysis. A Kruskal-Wallis rank-sum test was used to analyze differences between the distance traveled in E3 medium alone, E3 medium with neomycin, and E3 medium with both UoS-7692 and neomycin; pairwise comparisons were made using Dunn’s post hoc test. A 2-sample, 2-tailed *t* test was used to analyze the differences between the distances traveled in E3 medium and E3 medium containing UoS-7692. Plots and statistical analysis were conducted in R, version 3.5.0 (64).

#### In vivo hair-cell survival and ABR threshold shifts.

OHC survival in the cochleae of mice that received systemic injection with furosemide/kanamycin and single ear TTI of UoS-7692 or DMSO was analyzed by 2-way ANOVA with matched pairs design between cochleae from the same animal, followed by Šidák’s multiple-comparison test between injected and noninjected ears.

ABR threshold shifts from baseline were analyzed by 1-way ANOVA for click and individual pure-tone frequencies with Dunnett’s multiple-comparison test between each condition and either the 9-day DMSO condition or the 2-day kanamycin/furosemide-alone condition. Statistical analysis was performed using GraphPad Prism version 8.0.

### Study approval

Animals were raised following UK Home Office guidelines. All experiments were performed in accordance with the Home Office Animals (Scientific Procedures) Act 1986 and approved by the University of Sussex and University of Sheffield Animal Welfare Ethical Review Boards.

## Author contributions

EJK performed the zebrafish screens with the assistance of VNM, SB, and TTW. NKK tested the effects of compounds on K^+^ and MET currents in cochlear hair cells. SRK evaluated and quantified the protective properties of compounds in mouse cochlear cultures and analyzed GTTR-loading data, with the assistance of GPR and RTO. RJG and CDW tested and evaluated the protective properties of UoS-7692 in vivo. MD analyzed the structure and purity of the compounds. DMM and SJW tested the effects of UoS-7692 on the bactericidal properties of gentamicin. AVL compared compound similarity and provided visualization of the chemical screen data. RTO, SRK, and JCB provided statistical analysis of the data sets. The behavioral assays with zebrafish were performed by EJK. SRK organized and curated the data streams. VNM and CJK helped analyze and fit the electrophysiological data. NKK and SRK wrote preliminary drafts of the paper. SEW, CJK, and GPR conceived and directed the study. EJK, NKK, and SRK contributed equally to the work and are listed in alphabetical order. All authors contributed to writing the paper.

## Supplementary Material

Supplemental data

Supplemental data

## Version 1 03/18/2021

In-Press Preview

## Version 2 04/08/2021

Electronic publication

## Version 3 04/14/2021

Updated authorship note
