# Supplementary material for: Identification of a series of hair-cell MET channel blockers that protect against aminoglycoside-induced ototoxicity
Source: JCI Insight. 2021 Apr 8;6(7):e145704. doi: 10.1172/jci.insight.145704 (PMC8133782; doi:10.1172/jci.insight.145704)
Supplement: Supplemental data [file jciinsight-6-145704-s018.html]

The polar scatterplot shows all 10,240 compounds tested in zebrafish larvae for ability to protect against neomycin. Those in dark blue in the outer circle are classified as strong hair cell protectants, those in light blue are classified as weak hair cell protectants, those in grey did not protect hair cells and those in red were toxic to the zebrafish larvae. To view the compounds that protected zebrafish hair cells from gentamicin-induced damage click off "Zebrafish-All Compounds Neo" in the key. To view all compounds that protected mouse cochlear cultures (MCC) from gentamicin-induced damage click off "Zebrafish-All Compounds Neo" and "Zebrafish-Gent" in the key. To view the compounds that protected mouse cochlear cultures and did not cause hair-bundle damage (HBD) click off all the items in the key except "Mouse-Gent-w/o HBD" which should remain illuminated. To view the Life Chemical product ID for the compounds tested place the cursor over the spots in the polar plot.
