# Supplementary material for: Identification of a series of hair-cell MET channel blockers that protect against aminoglycoside-induced ototoxicity
Source: JCI Insight. 2021 Apr 8;6(7):e145704. doi: 10.1172/jci.insight.145704 (PMC8133782; doi:10.1172/jci.insight.145704)
Supplement: Supplemental data [file jciinsight-6-145704-s019.pdf]

Supplemental Methods

Qualitative assessment of zebrafish neuromasts for hair-cell protection

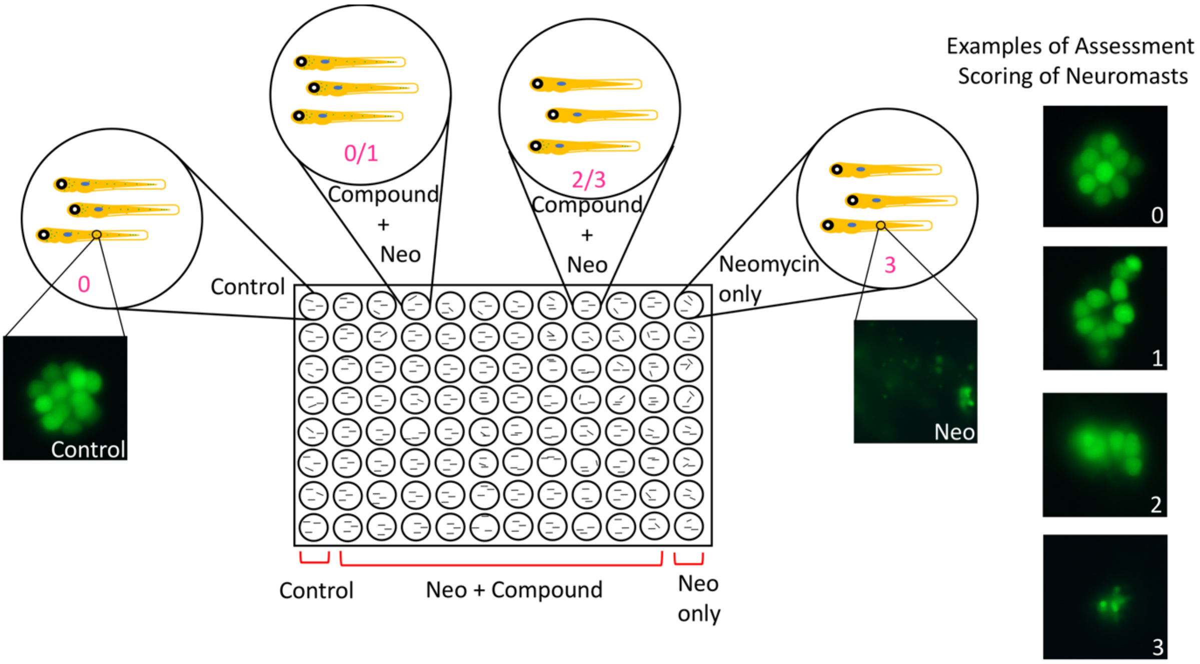

Figure illustrating the methods used to qualitatively assess screening plates. Examples of neuromast scores for larvae treated with compound and aminoglycoside are shown on the right while examples are shown in the main schematic for control (left) and aminoglycoside only (right) treated neuromasts. All images are from 4th neuromast in the posterior lateral line.

Wells were observed from left to right during screening of a plate, a process that took between 20-30 minutes. Posterior lateral line (trunk) neuromasts in three fish in each well were observed and wells were given an overall score (examples scores shown in pink in schematic) as follows:

0 = full protection with no dead hair cells (i.e., with no hair-cell fragments) observed in any of the trunk neuromasts of any fish;
0/1 = protection with hair-cell fragments visible in the trunk neuromasts of some fish;
1 = protection with hair-cell fragments visible in the neuromasts of all fish
1/2 = weak protection with less than 5 hair cells present in the neuromasts of some fish and the majority of hair cells present in the other fish;
2 = weak protection with fewer than 5 hair cells present in the trunk neuromasts of all fish;
2/3 = no protection with one or two hair cells present in the trunk neuromasts of some fish;
3 = no protection with no hair cells or only hair-cell fragments visible in the trunk neuromasts of all fish.

Compounds were considered protective if the well scored 0, 0/1 or 1.

Supplemental Results

Supplemental Figure 1: Interactive Polar Plot for Diversity Set Screen

https://sussex-neuroscience.github.io/Identification-of-a-series-of-hair-cell-MET-channel-blockers/Supplemental%20Data%20Polar%20Scatterplot.html

The polar plot shows all 10,240 compounds tested in zebrafish larvae for ability to protect against neomycin. Those in dark blue in the outer circle are classified as strong hair cell protectants, those in light blue are classified as weak hair cell protectants, those in grey did not protect hair cells and those in red were toxic to the zebrafish larvae. To view the compounds that protected zebrafish hair cells from gentamicin-induced damage click off "Zebrafish-All Compounds Neo" in the key. To view all compounds that protected mouse cochlear cultures (MCC) from gentamicin-induced damage click off "Zebrafish-All Compounds Neo" and "Zebrafish-Gent" in the key. To view the compounds that protected mouse cochlear cultures and did not cause hair-bundle damage (HBD) click off all the items in the key except "Mouse-Gent-w/o HBD" which should remain illuminated. To view the Life Chemical product ID for the compounds tested place the cursor over the spots in the polar plot.

Supplemental Figure 2: Prestin labelling of OHCs in ears from UoS-7692 protected and non-protected ears of mice following furosemide/kanamycin exposure

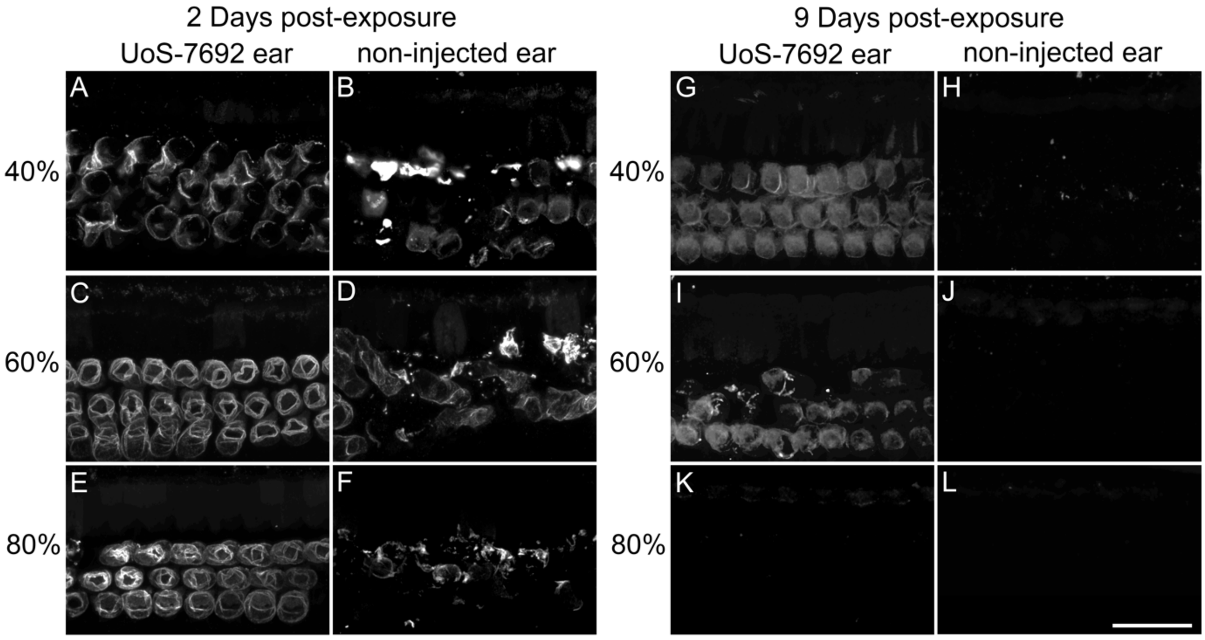

Supplemental Figure 2: Prestin staining of OHCs in regions 40% (A, B, G, H), 60% (C, D, I, J) and 80% (E, F, K, L) from the apical end of the cochlea in mice that received UoS-7692 protection (A, C, E, G, I, K) in one ear and no protection (B, D, F, H, J, L) in the opposite ear followed by exposure to systemic furosemide/kanamycin. Animals were maintained for either 2 (A-F) or 9 (G-L) days. After 2 days, normal OHC bodies are abundant in UoS-7692 treated ears in all regions (A, C, E) whereas in non-protected cochleae, very few intact OHC hair bodies are seen, particularly at the 80% region. After 9 days OHC bodies are intact in the 40% region in the UoS-7692 treated cochleae, some remain in the 60% region but all are lost from the 80% region. In non-protected cochleae, OHC bodies are absent from all regions after 9 days. Bar in L = 20 µm.

Supplemental Figure 3: Furosemide treatment does not affect ABR thresholds.

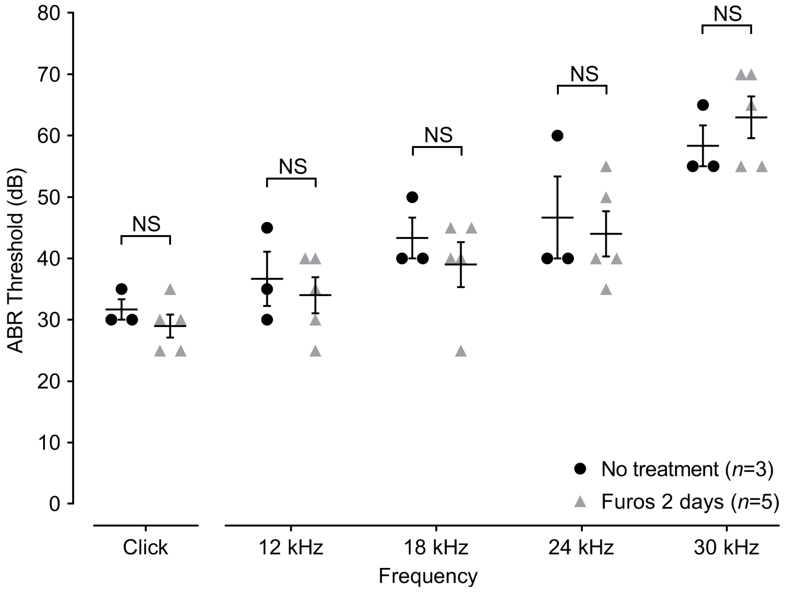

Supplemental Figure 3: ABR thresholds measured in mice either receiving no treatment (black circles) or 2 days following systemic injection with 15 µL/g furosemide (grey triangles). There is no difference in ABR threshold in response to click stimulus or pure-tone frequencies as a result furosemide treatment (Two-tailed t tests at click and individual pure-tone frequencies). NS $P > 0.05$. *N* numbers are shown in the key. Error bars show SEM.
